# Birth weight in relation to endometrial and breast cancer risks in Swedish women

**DOI:** 10.1038/sj.bjc.6603504

**Published:** 2006-12-05

**Authors:** M Löf, S Sandin, L Hilakivi-Clarke, E Weiderpass

**Affiliations:** 1 Department of Medical Epidemiology and Biostatistics, Karolinska Institute, PO 281, SE-171 77, Stockholm, Sweden; 2 Department of Oncology, Georgetown University, Washington, DC 20057, USA; 3 The Cancer Registry of Norway, N-0310, Montebello, Oslo, Norway

**Keywords:** endometrial cancer, breast cancer, birth weight

## Abstract

An examination of birth weight in a Swedish cohort study of 38 566 women showed no significant association between birth weight and endometrial cancer, but supported a protective role for low birth weight for premenopausal breast cancer.

Evidence for a positive association between birth weight and risk of adult hormone-dependent cancers is limited primarily to breast cancer. Only one study has investigated whether there is an association between birth weight and endometrial cancer (McCormack *et al*, 2005). During a follow-up of 41 years, women with higher birth weights had a reduced risk of endometrial cancer (McCormack *et al*, 2005). Although current evidence strongly suggests that high birth weight increases premenopausal breast cancer risk (Michels and Xue, 2006), several of these studies investigated women born in the 1910–1940s (Andersson *et al*, 2001; Titus-Ernstoff *et al*, 2002; dos Santos Silva *et al*, 2004; McCormack *et al*, 2005). These women were born and grew up during or just after World War I and II, when their nutritional status *in utero* and during childhood was probably different from that among women born later. Further, the role for low birth weight for breast cancer risk is not fully worked out. Adjustment for adult body mass index (BMI) is highly relevant, as it is associated with birth weight (Oken and Gillman, 2003) and also affects adult breast cancer risk (Huang *et al*, 1997). Also, adult BMI is a strong risk factor for endometrial cancer (Amant *et al*, 2005). We have investigated whether low birth weight is associated with endometrial and breast cancer risks in a prospective cohort of Swedish women born during 1942–1962, adjusting for adult BMI.

## MATERIALS AND METHODS

The study cohort consisted of 49 259 Swedish women in the Women's Lifestyle and Health study (Kumle *et al*, 2002). The mean age at baseline in 1991 was 39 years and 9% of the women were postmenopausal. Participants reported weight and height at enrolment, and their own birth weight (<2.5 kg, 2.5–3 kg, or >3 kg) using a questionnaire. Complete follow-up was achieved from linkages with nationwide health registers through December 2003, yielding 73 cases of endometrial cancer, and 657 cases of breast cancer. We excluded 10 693 women from the initial cohort because of: breast or endometrial cancer before enrolment (*n*=253), emigration before the start of the follow-up (*n*=16), hysterectomy (*n*=1501), twins (*n*=1154), and with missing information on birth weight or adult BMI (*n*=7769). Thus, the final analysis was conducted on 38 566 women. The study was approved by the ethical committee at the University of Uppsala and the Karolinska Institute.

The risks of endometrial and breast cancers were analysed by fitting of Cox regression models. One crude model including only the birth weight variable was fitted for both endometrial and breast cancer. A second model including BMI at enrolment was fitted for both cancer forms. We also fitted models including the following covariates: parity, age at first birth, total months of breastfeeding, and family history of breast cancer (mother or sister). For all models, the attained age at follow-up was used as time scale and stratified by 5-year birth cohorts. Hazard ratios (HR) were declared statistically significant when the associated two-sided 95% Wald-type confidence interval did not cover the value one. This corresponds to a two-sided 5% level of significance. All statistical analyses were carried out using the SAS software version 9.1, (SAS Institute Inc., Cary, NC, USA).

## RESULTS

The baseline characteristics of the 38 566 women in this study are presented in Table 1. Thus, 5% (1975) of the 38 566 women reported birth weights <2.5 kg, whereas 20% (7877) and 75% (28 714) reported birth weights 2.5–3 kg and >3 kg, respectively. Five per cent (four) of the endometrial cancer cases reported birth weights <2.5 kg, whereas 15% (11) reported birth weights 2.5–3 kg, and 80% (58) reported birth weights >3 kg. The corresponding figures for the breast cancer cases were 4% (23), 22% (144), and 74% (490) for birth weights <2.5 kg, 2.5–3 kg, and >3 kg, respectively. Fifty-six per cent (41) of the endometrial cancer cases were normal weight (BMI<25 kg/m^2^), whereas 28% (20) were overweight (BMI 25–30 kg/m^2^), and 16% (12) were obese (⩾30 kg/m^2^). The corresponding figures for the breast cancer cases were: 76% (501) normal weight, 19% (123) overweight, and 5% (33) obese. When compared to the 38 566 women who were included in the analysis, the distribution of the baseline characteristics were similar for the 7769 women who were excluded from the study owing to missing values of birth weight or adult BMI (data not shown).

**Figure 1:**
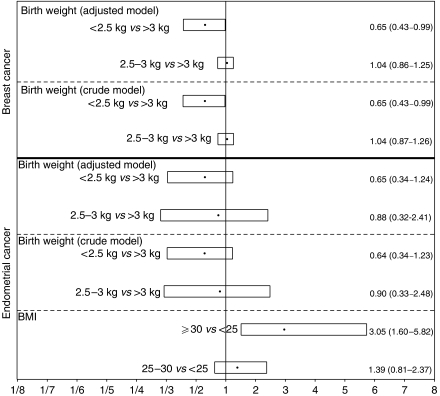
Estimated HR (black dots) with associated two-sided 95% Wald confidence intervals (boxes) for comparison of two categories, for example women with a birth weight <2.5 kg compared to women with a birth weight >3 kg. The adjusted model for both breast and endometrial cancer include the covariates birth weight (<2.5kg, 2.5–3kg and >3 kg) and adult BMI (<25, 25–30 and ⩾30 kg/m^2^) both as categorical covariates. Additionally, the HR estimates for endometrial cancer risk for normal weight women (BMI<25) compared to overweight women (BMI 25–30) and obese women (BMI⩾30), respectively, provided by the adjusted model are shown. All models were fitted stratified by 5-year birth cohorts utilising attained age as time scale.

The HR for endometrial and breast cancer risk for different birth weight categories are shown in Figure 1. The crude HR for endometrial cancer risk for women with low birth weight (<2.5 kg) compared to women with normal to high birth weight (>3 kg) was 0.6, and not statistically significant. Adjusting for adult BMI did not modify the HR. The adjusted model also provided HR estimates for endometrial cancer risk for different BMI categories (BMI<25, BMI 25–30, and BMI⩾30 kg/m^2^). Obese women (BMI⩾30 kg/m^2^) had a statistically significant increased risk of endometrial cancer (HR: 3.1, 95% CI: 1.6–5.8) compared to normal weight women (BMI<25).

The crude HR for breast cancer risk, when comparing women with low birth weight (<2.5 kg) to women with the highest birth weight (>3 kg) was 0.65 (95% CI: 0.43–0.99). The results were not affected by adjustment for adult BMI.

The HR for endometrial and breast cancer reported in Figure 1 were not affected by adjustment for other baseline covariates like parity, age at first birth, total months of breastfeeding, or family history of breast cancer (mother or sister) (data not shown).

## DISCUSSION

We found no evidence of an association between low birth weight, and reduced endometrial cancer risk, although our data supported earlier findings that a higher birth weight increases breast cancer risk. The results also suggest that low birth weight is protective for breast cancer. Adult BMI did not modify the effect of a low birth weight on endometrial or breast cancer risk.

Strengths of this study are the prospective design, and the complete follow-up. One major limitation is that we only had three categories of birth weight, and the highest category included infants of normal as well as of high birth weight. Earlier studies have suggested that birth weights above 4000 g increase breast cancer risk (Sanderson *et al*, 1996; McCormack *et al*, 2003, 2005; Mellemkjaer *et al*, 2003; Ahlgren *et al*, 2004; dos Santos Silva *et al*, 2004), while increased (Innes *et al*, 2000; Mellemkjaer *et al*, 2003), or reduced (Michels *et al*, 1996; Michels *et al*, 2006) risks have been reported for low birth weight. Results of this study indicate that among women born in the 1940–1960s, a low birth weight is associated with reduced breast cancer risk. The results suggest that the earlier reported link between birth weight, and breast cancer in women born in the early 1900s is also relevant for women born in the mid-1900s.

Another limitation of the study was that we could not adjust for gestational age, which has been shown to strengthen the positive association between birth weight and breast cancer risk (McCormack *et al*, 2003). Some misclassification of birth weight (Troy *et al*, 1996; Andersson *et al*, 2000) is unavoidable but likely nondifferential and thus, would have led to an underestimation of the true association.

The inconsistency between this study and the only other study on endometrial cancer (McCormack *et al*, 2005) may be due to the small number of cases in both. Still, although we had only 73 cases we were able to confirm earlier findings that obesity increases endometrial cancer risk (Amant *et al*, 2005). Other possible explanations include that women in our study were born approximately 30 years later than women in the study by McCormack *et al* (2005), our women were younger than 50 years of age, whereas their women were mostly postmenopausal, or that we focused on investigating the effect of a low birth weight, while they examined the impact of a high birth weight on endometrial cancer.

In conclusion, no association between birth weight and endometrial cancer risk was found, whereas a low birth weight was suggested to reduce the risk of developing breast cancer among women born in the 1940–1960s.

## Figures and Tables

**Table 1:** Baseline characteristics of the women of the study (*n*=38 566)

	**Birth weight (kg)**		
	***<*2.5** **(*n=*1975)**	**2.5–3 (*n*=7877)**	**>3 (*n*=28 714)**
*BMI at enrolment* (*kg/m*^2^)			
Median	23	23	23
Quartile 1	21	21	21
Quartile 3	25	25	25
Range	16–48	15–67	13–65
			
*Age at enrolment* (*years*)			
Median	39	39	38
Quartile 1	34	34	34
Quartile 3	44	44	43
Range	29–49	29–49	29—49
			
*Parity at enrolment*	Number of women (%)		
0	328 (17)	1177 (15)	3783 (13)
1	279 (14)	1276 (16)	4365 (15)
2	843 (43)	3334 (42)	12555 (44)
3	383 (19)	1561 (20)	6127 (21)
⩾4	142 (7)	529 (7)	1884 (7)
